# Laser‐Defined States and Hydration‐Driven Relaxation in PEDOT:PSS

**DOI:** 10.1002/smll.74614

**Published:** 2026-07-28

**Authors:** Chanwoo Kim, Soonwook Jung, Won‐Seok Kim, Sanghun Lee, Songkil Kim, Habeom Lee

**Affiliations:** ^1^ School of Mechanical Engineering Pusan National University Busan Republic of Korea

**Keywords:** conductive polymer, hydration, laser processing, PEDOT:PSS, state switching

## Abstract

Conductive polymers such as PEDOT:PSS undergo complex structural and electronic changes under external stimuli, yet their state evolution under sequential thermal and hydration processes remains poorly understood. Here, we investigate laser–water interactions in PEDOT:PSS from the perspective of material state evolution and relaxation rather than simple modification and erasure. Using a mechanically interlocked PEDOT:PSS configuration that remains stable under repeated laser irradiation and hydration, we systematically examine how laser‐defined material states evolve upon subsequent water exposure. Continuous‐wave laser irradiation induces a distinct non‐carbonized blue optical transition that is clearly distinguishable from irreversible carbonization. Upon water exposure, this laser‐defined state exhibits reversible optical and morphological changes, while the extent of relaxation strongly depends on the prior laser irradiation conditions. Correlative analyses of surface and internal morphology, molecular structure, and electrical response reveal that hydration‐driven relaxation does not necessarily restore the pristine state but instead proceeds along pathways determined by the laser‐induced configuration. These findings demonstrate that water acts as an active mediator of state relaxation rather than a simple reset mechanism. This work provides a framework for understanding history‐dependent state evolution in hydration‐sensitive conductive polymers under sequential laser and environmental stimuli.

## Graphical abstract

Laser irradiation defines metastable states in PEDOT:PSS, while water drives hydration‐mediated relaxation rather than simple erasure. This sequential laser–water process enables reversible optical rewriting with history‐dependent structural and electrical responses.

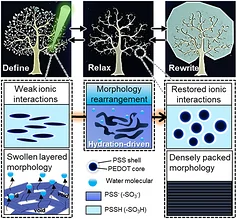

## 1 Introduction

PEDOT:PSS is widely used as a conductive polymer in thermoelectric applications [1, 2, 3, 4], photovoltaics [5, 6, 7, 8], and flexible electronics [9, 10, 11, 12], where its electrical and optical properties are highly sensitive to environmental conditions. This sensitivity originates from the intrinsically coupled nature of the PEDOT:PSS network, in which electronic transport through PEDOT‐rich domains is strongly influenced by ionic interactions with PSS and by the presence of absorbed water [13, 14, 15, 16, 17, 18]. Changes in hydration can modulate Coulombic interactions between PEDOT^+^ and PSS^−^ [19], alter nanoscale domain morphology, and consequently affect charge transport [20, 21], optical appearance [22, 23], and mechanical behavior [24, 25]. As a result, PEDOT:PSS often exhibits pronounced variations in conductivity, transparency, and surface structure in response to moisture, thermal history, or processing conditions [26, 27, 28, 29, 30, 31]. Such pronounced environmental sensitivity indicates that the properties of PEDOT:PSS are highly susceptible to external stimuli.

Among the various external stimuli applied to PEDOT:PSS, laser irradiation represents a uniquely localized and intense thermal perturbation. Laser exposure can generate steep temperature gradients on sub‐millimeter length scales, leading to local dehydration, polymer rearrangement, and in some cases, carbonization [32, 33, 34]. Such highly localized stimulation distinguishes laser processing from more homogeneous treatments such as thermal annealing or solvent exposure. Consequently, laser patterning has been widely adopted as a versatile tool for defining conductive features, modifying surface morphology, or creating functional gradients in PEDOT:PSS‐based devices [35, 36, 37, 38, 39, 40, 41, 42]. However, many laser‐based approaches to PEDOT:PSS have focused on irreversible patterning or modification, including chemical degradation and carbonization [43] under high irradiation doses. As a result, the possibility that laser irradiation may also define non‐destructive, stimulus‐specific material states has received comparatively less attention.

In stimulus‐responsive polymer systems, reversibility is often implicitly assumed to correspond to a complete return to the original material state. This assumption is also reflected in previous studies on PEDOT:PSS, where exposure to water or humid environments has sometimes been treated as a practical route to partially reverse prior processing‐induced changes, particularly when macroscopic properties appear to recover [44, 45, 46, 47]. However, because hydration actively modifies internal interactions and mobility within ionic polymer networks, macroscopically observed recovery does not necessarily imply restoration of the original material state, highlighting the need to distinguish apparent recovery from true relaxation of the state. Addressing this question is complicated by the intrinsic instability of PEDOT:PSS under hydrated conditions, which constrains systematic investigation of laser–water interactions.

In many commonly used configurations, PEDOT:PSS films undergo swelling, delamination, or partial dissolution upon water exposure, making it difficult to repeatedly probe the same region before and after hydration [48, 49, 50]. As a result, experimental observations are often limited to either dry, laser‐processed states or fully hydrated conditions, with little access to intermediate relaxation processes. Overcoming this limitation requires a mechanically robust and water‐tolerant configuration that preserves film integrity during repeated laser irradiation and hydration, thereby enabling laser‐defined regions to be interrogated throughout the entire laser–water sequence.

In this work, we investigate laser–water interactions in PEDOT:PSS under sequential stimulation, focusing on how laser‐defined material states evolve upon subsequent hydration rather than assuming simple modification or erasure. To enable this analysis, we employ a mechanically interlocked PEDOT:PSS configuration that remains stable under repeated laser irradiation and water exposure, allowing laser‐defined regions to be repeatedly readdressed and interrogated. Through correlative analyses of optical appearance, surface and internal morphology, molecular structure, and electrical response, we show that hydration does not simply restore the pristine state but instead drives relaxation along pathways determined by the prior laser‐defined configuration. These findings reveal that water functions not as a passive resetting agent but as an active mediator of state relaxation. This perspective provides a framework for understanding stimulus‐history‐dependent behavior in hydration‐sensitive conductive polymers.

## 2 Results and Discussion

**FIGURE 1:**
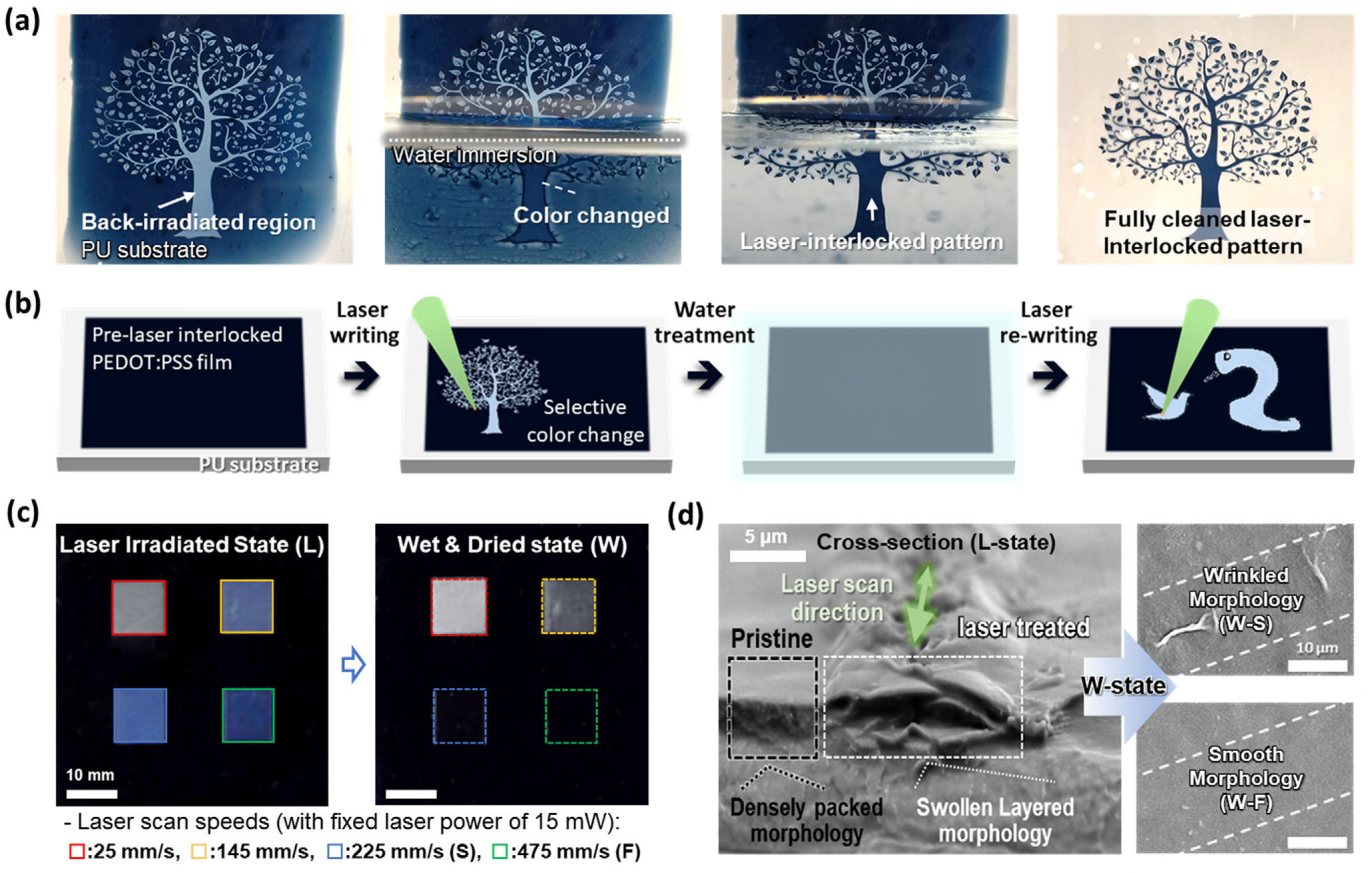
Laser‐water‐mediated optical and morphological state transitions in interlocked PEDOT:PSS films. (a) Optical images showing a reversible blue optical transition in laser‐patterned PEDOT:PSS films upon water exposure and drying. (b) Schematic illustration of the laser writing–water treatment–laser rewriting process, demonstrating selective optical modulation and reconfiguration. (c) Optical images of L‐state and W‐state regions under different laser scanning speeds, highlighting reversible blue‐transition states and irreversible carbonized regions. (d) Cross‐sectional SEM image of an L‐state region showing a locally swollen and layered morphology, together with surface SEM images corresponding to the W‐state, revealing scan‐speed‐dependent morphological evolution into wrinkle‐like (W‐S) or smooth (W‐F) structures.

The reversible optical state switching reported in this study was first observed during continuous‐wave laser patterning of PEDOT:PSS films. Under specific irradiation conditions, selectively patterned regions exhibited a pronounced blue optical transition that was clearly distinguishable from laser‐induced carbonization (Figure S1). As shown in Figure 1a, laser‐back‐irradiation‐induced patterned PEDOT:PSS films displayed localized blue‐transitioned regions that reverted to the characteristic color of pristine PEDOT:PSS upon water exposure. Notably, the patterned films remained strongly adhered to the substrate even under fully hydrated conditions, indicating that the blue optical transition does not originate from irreversible chemical degradation but instead arises from a reversible, water‐responsive structural modification further supported by microscopic images shown in Figure S2.

To reproducibly isolate this behavior, pristine PEDOT:PSS was uniformly deposited onto a polyurethane (PU) substrate and subjected to laser back‐scanning followed by water rinsing. This process selectively removed loosely bound PEDOT:PSS components, resulting in a mechanically interlocked PEDOT:PSS film firmly anchored to the PU substrate [35, 39]. This interlocked film is hereafter defined as the pristine state. Subsequent laser writing on the pristine state enabled spatially selective optical modulation, and the written patterns could be erased and reconfigured through water treatment and repeated laser irradiation, as illustrated schematically in Figure 1b.

Depending on the laser irradiation conditions, three distinct laser‐induced responses were observed (Figure 1c): (i) an irreversible carbonized state, (ii) a mixed state exhibiting partial carbonization accompanied by a blue optical transition, and (iii) a non‐carbonized blue‐transition state. In this work, the term laser‐defined state (L‐state) specifically refers to the non‐carbonized, blue‐transition state that recovers its optical appearance after water treatment. Within the L‐state regime, two representative conditions were further defined based on laser scanning speed with fixed laser power of 15 mW: a slow‐scan condition (L‐S, 225 mm s^−1^) and a fast‐scan condition (L‐F, 475 mm s^−1^), which serve as key comparative cases throughout the following analysis. A processing window defining these regimes, including the carbonized state, L‐state, and no‐change region, is summarized in Figure S3, providing practical guidelines for future scalable implementation.

To elucidate the structural origin of the blue optical transition, cross‐sectional SEM analysis was performed on representative L‐state regions (Figure 1d). The laser‐irradiated areas exhibited a locally swollen and layered morphology, clearly distinguishable from the densely packed structure of pristine PEDOT:PSS. The induced surface expansion was aligned with the laser scan direction and accompanied by internal structural rearrangement across the film thickness. Such laser‐induced morphological modulation alters the local optical environment of the film, giving rise to angle‐dependent optical responses consistent with structural coloration attributed to thin‐film interference (Figure S4) [51, 52].

After water exposure and drying, the resulting morphology is hereafter defined as the water‐relaxed state (W‐state). Following hydration, the laser‐induced morphologies evolve in a scanning‐speed‐dependent manner. Specifically, L‐S regions developed wrinkle‐like surface features (W‐S), whereas L‐F regions relaxed into comparatively smooth morphologies (W‐F), reflecting distinct structural relaxation pathways. This behavior is clearly distinguishable from the carbonized features that largely remain stable after water treatment in the mixed state (Figure S5). These observations demonstrate that sequential laser irradiation and water treatment enable reversible modulation of optical appearance and surface morphology in interlocked PEDOT:PSS films.

**FIGURE 2:**
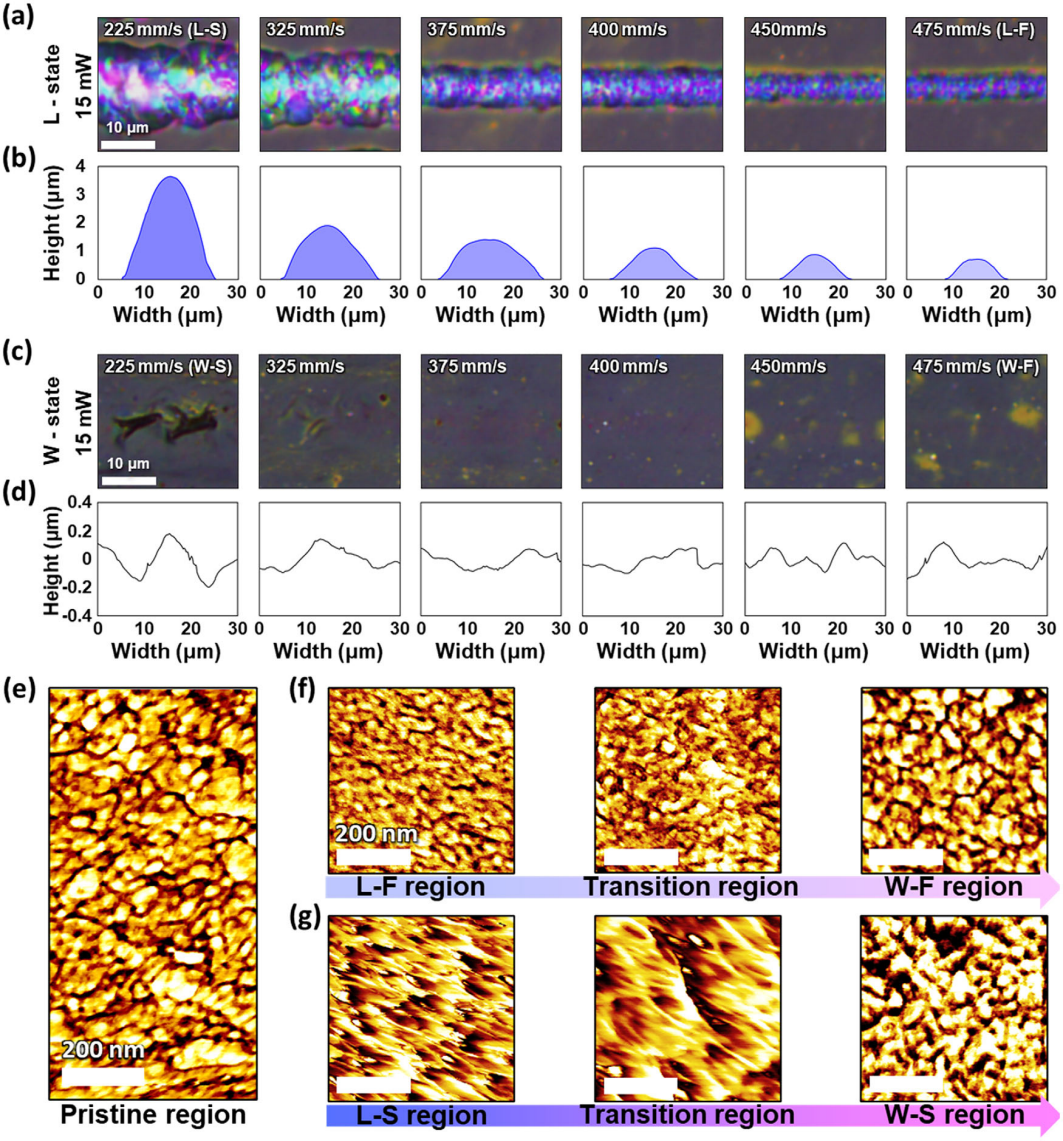
Micro‐ and nanoscale morphological evolution during L–W state transition. (a) Optical microscopy images of L‐state regions fabricated at a fixed laser power of 15 mW with varying scanning speeds. (b) Cross‐sectional height profiles of the L‐state regions, revealing scan‐speed‐dependent surface swelling behavior. (c) Optical microscopy images of the corresponding W‐state regions under identical laser conditions. (d) Cross‐sectional height profiles of the W‐state, showing relaxation behavior that depends on the initial laser‐induced morphology. (e–g) AFM phase images illustrating domain‐scale structural evolution during the L–W state transition for representative L‐S (225 mm s^−1^) and L‐F (475 mm s^−1^) conditions. Bright and dark contrasts correspond to PEDOT‐rich and PSS‐rich domains, respectively [66].

The morphological features of the laser‐irradiated (L) and water‐treated (W) states were examined to clarify the structural origin of the optical state switching. Figure 2a presents optical microscopy images of L‐state regions fabricated at a fixed laser power of 15 mW while varying the scan speed from 225 to 475 mm s^−^^1^. Across the entire scan‐speed range, distinct blue optical contrast was consistently observed, clearly differentiating the laser‐processed regions from the pristine film. As discussed above, the blue optical transition is accompanied by laser‐induced morphological restructuring. Cross‐sectional height profiles measured by AFM (Figure 2b) reveal parabolic surface features whose shape is consistent with the Gaussian intensity distribution of the TEM_00_ laser mode [53]. The maximum height and lateral width of the swollen structures decrease with increasing scan speed, reflecting the reduced photothermal energy delivered to the PEDOT:PSS film. This result demonstrates that the geometric characteristics of the L‐state can be continuously tuned by controlling laser scanning parameters. SEM images (Figure S6) further resolve the surface morphology of the L‐state. With increasing scan speed, the surface morphology transitions from undulating features observed under the L‐S condition (225–325 mm s^−^^1^) to dome‐like structures with more uniform width and height under the L‐F condition (375–475 mm s^−^^1^). These observations indicate a transition from highly undulating surface deformation at high thermal dose to more localized and uniform dome‐like swelling as the local thermal dose decreases. This trend is consistent with a reduced local thermal dose at higher scan speeds, which limits the spatial extent of laser‐induced deformation [54, 55].

Following water exposure and drying, the surface morphology undergoes a distinct rearrangement, defining the W‐state. Figure 2c shows optical images of W‐state regions corresponding to each laser condition. After hydration, the laser‐induced surface morphologies formed in the L‐state undergo partial relaxation across all samples. Closer examination reveals a scan‐speed‐dependent relaxation behavior: regions processed at lower scan speeds (225–325 mm s^−^^1^) retain residual wrinkled surface features, whereas those processed at higher scan speeds (≥375 mm s^−^^1^) relax toward smooth surfaces comparable to the pristine film, as confirmed by AFM height profiles (Figure 2d) and AFM topography images (Figure S7). Additional optical images, height profiles, and AFM phase images (Figure S8) provide further insight into the intermediate stages of water‐induced relaxation. When one side of a laser‐treated region is exposed to water, capillary‐driven infiltration leads to partial hydration extending toward the opposite side, creating a spatial gradient in hydration. Within this transition region, the surface morphology exhibits features intermediate between the L‐ and W‐states, providing a snapshot of the ongoing relaxation process under hydrated conditions.

AFM phase images (Figure 2e–g) provide direct insight into domain‐scale structural evolution during the L–W transition. Laser irradiation transforms the originally granular PEDOT:PSS domains into elongated fibrillar structures, defining the L‐state, with increasing alignment at lower scan speeds. This behavior is consistent with laser‐induced phase separation and a reduction in electrostatic interactions between PEDOT and PSS, analogous to structural transitions reported for solvent‐screened or acid‐treated conjugated polymer systems [56, 57, 58, 59]. Notably, the fibrillar domains exhibit preferential orientation along the laser scanning direction, suggesting that the continuous‐wave laser‐induced thermal field [60, 61, 62] promotes directional mass transport and polymer rearrangement (Figure S9) [63], distinct from the isotropic phase rearrangement induced by non‐directional heat fields [39, 64, 65]. Upon hydration, the laser‐defined domain structures undergo partial relaxation, leading to blurred domain boundaries and a gradual loss of fibrillary order in the transition regions. In the fully developed W‐state, the domains reorganize into granular morphologies, indicating recovery of PEDOT–PSS electrostatic interactions. While the W‐F regions closely resemble the pristine morphology, the W‐S regions retain larger granular features, reflecting incomplete relaxation from the highly deformed L‐state. Overall, these observations demonstrate that laser irradiation induces scan‐speed‐dependent domain‐level rearrangements in PEDOT:PSS, while subsequent hydration drives relaxation pathways that depend on the initial laser‐defined state.

**FIGURE 3:**
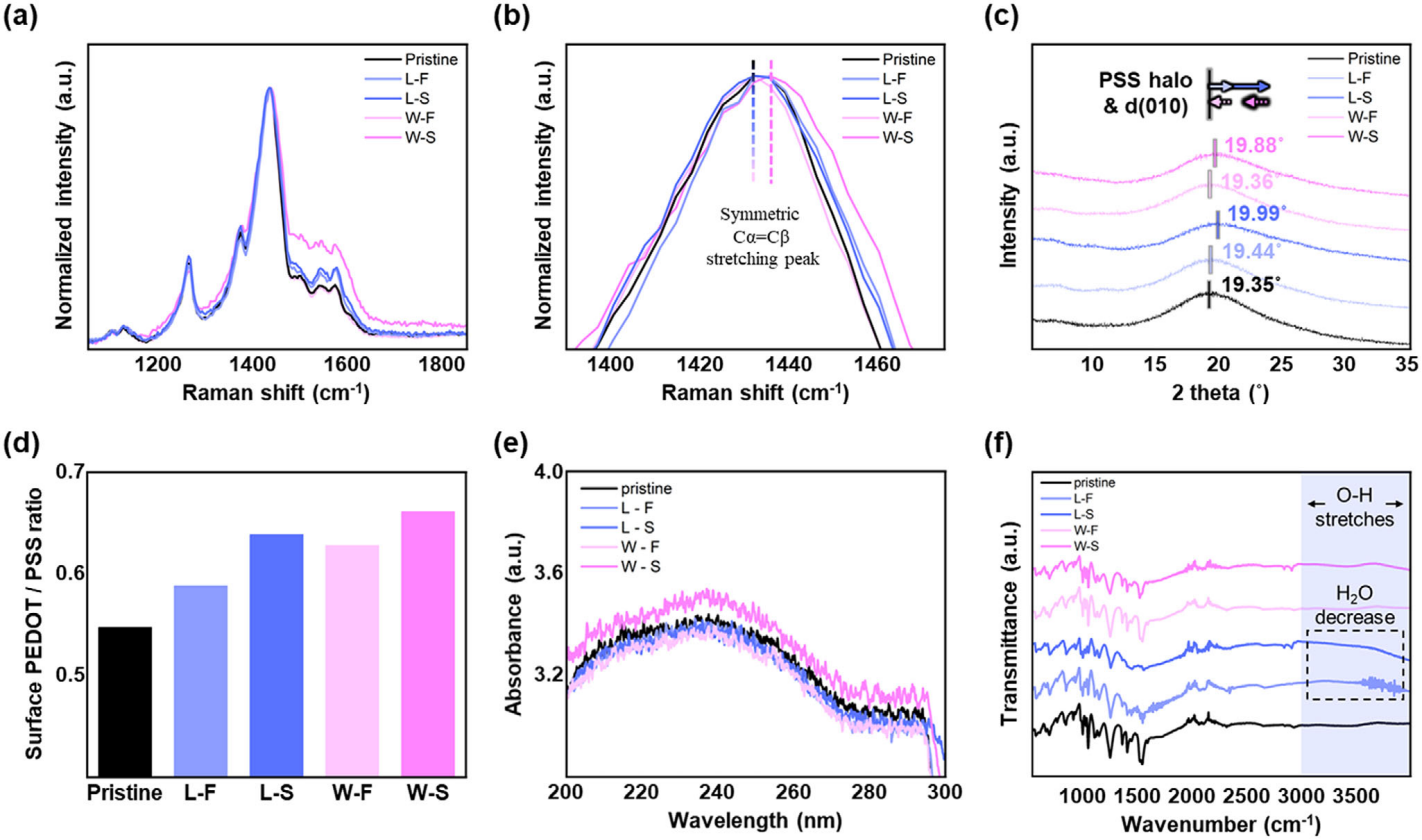
Spectroscopic and structural characterization during the L–W state transition (a) Raman spectra of pristine, L‐S, L‐F, W‐S, and W‐F PEDOT:PSS films. (b) Enlarged Raman spectra showing the PEDOT Cα = Cβ stretching region (1420–1450 cm^−^^1^). (c) XRD patterns of pristine, L‐S, L‐F, W‐S, and W‐F PEDOT:PSS films. (d) Surface PEDOT‐to‐PSS ratio determined by XPS analysis for pristine, L‐S, L‐F, W‐S, and W‐F PEDOT:PSS films. (e) UV–vis absorption spectra in the 200–300 nm range. (f) FT‐IR spectra of pristine, L‐S, L‐F, W‐S, and W‐F PEDOT:PSS films.

Structural and chemical analyses were performed on pristine, L‐state, and W‐state PEDOT:PSS films to elucidate the molecular and structural origins of the laser‐water‐induced state switching. Figure 3a,b present the Raman spectra, in which the observed vibrational features are primarily associated with PEDOT chains, as PSS exhibits comparatively weak Raman scattering [67]. Upon laser irradiation, both L‐S and L‐F samples exhibit increased Raman intensities in the 1480–1600 cm^−1^ region, which are attributed to enhanced asymmetric Cα = Cβ stretching vibrations and a possible increase in the effective doping level of the PEDOT chains [67, 68, 69]. In addition to this intensity enhancement, a characteristic symmetric Cα = Cβ stretching band, commonly associated with the conjugated backbone structure of PEDOT, is observed in the 1420–1450 cm^−1^ range. The slight blueshift of this band indicates increased backbone linearity, while its broadened bandwidth suggests a more heterogeneous distribution of conjugated domains [70]. Collectively, the Raman response implies that laser irradiation alters the configurational state of PEDOT chains while maintaining their intrinsic chemical structure. Following water treatment and subsequent drying, the asymmetric and symmetric Cα = Cβ stretching peaks in W‐F largely revert toward their pristine positions and intensities, indicating substantial relaxation of the laser‐induced spectral changes. In contrast, W‐S retains intensified and blueshifted Cα = Cβ stretching features, reflecting a persistent modification of the PEDOT chain configuration. Such spectral behavior in W‐S is commonly associated with increased aggregation of PEDOT‐rich domains [39], which is consistent with the nanoscale morphological features observed in the corresponding AFM images.

To probe whether these conformational changes are accompanied by modifications in intermolecular packing, x‐ray diffraction(XRD) analysis was performed (Figure 3c). The pristine PEDOT:PSS film exhibits a diffraction peak at 2θ = 7.0° (*d* = 12.6 Å), which is assigned to the d_(100)_ lamellar stacking reflecting the periodic separation between PEDOT and PSS [59]. In addition, a broad diffraction feature centered around 2θ ≈ 19.35° is observed, arising from the overlapping contributions of the amorphous PSS halo at 2θ ≈ 19.5° (*d* = 4.5 Å) and the PEDOT d_(010)_ π–π stacking at 2θ ≈ 25.3° (*d* = 3.5 Å) [59, 71, 72]. This broad peak indicates the coexistence of ordered PEDOT stacking and disordered PSS phases within the pristine film. Following laser irradiation, the d_(100)_ lamellar peak shifts slightly toward lower angles, appearing at 2θ = 6.7° (*d* = 13.2 Å) for L‐S and 2θ = 6.8° (*d* = 13.0 Å) for L‐F, indicating an expansion of lamellar spacing within PEDOT‐rich domains. Concurrently, secondary diffraction peaks corresponding to d_(200)_ stacking emerge at 2θ = 10.1° (*d* = 8.7 Å) for L‐S and 2θ = 10.9° (*d* = 8.1 Å) for L‐F, suggesting enhanced lamellar ordering and the development of more well‐defined stacking periodicity [59]. In contrast, the broad diffraction feature associated with overlapping PSS halo and PEDOT d_(010)_ π–π stacking becomes less intense and shifts toward higher angles, appearing at 2θ = 19.99° for L‐S and 2θ = 19.44° for L‐F. This behavior reflects tighter π–π stacking and laser‐induced morphological rearrangement within the PEDOT:PSS matrix [72], with a clear dependence on laser scanning speed. Overall, these XRD features indicate thermally driven densification and ordering of PEDOT‐rich domains upon laser irradiation.

In the W‐state, the secondary d_(200)_ reflections diminish in both W‐S and W‐F, indicating a partial relaxation of the higher‐order lamellar stacking induced by laser irradiation. Meanwhile, the primary d_(100)_ peaks shift further toward lower angles, appearing below 2θ = 5° (*d* = 17.6 Å) for W‐S and at 2θ = 6.3° (*d* = 14.0 Å) for W‐F, reflecting an expansion of the lamellar spacing upon water treatment. In the higher‐angle region, the broad diffraction feature associated with overlapping PSS halo and PEDOT d_(010)_ π–π stacking shifts toward lower angles, nearly returning to the pristine position in W‐F (2θ = 19.36°), while remaining shifted to a higher angle in W‐S (2θ = 19.88°) relative to the pristine film. Taken together, these observations suggest that laser‐induced densification of inter‐chain packing in PEDOT‐rich domains is substantially relaxed in W‐F, whereas a portion of the compact molecular packing persists in W‐S even after water‐induced morphological rearrangement.

Surface compositional changes in PEDOT:PSS during state switching were examined using XPS and UV–vis spectroscopy to distinguish between chemical modification and spatial redistribution of PEDOT and PSS. Figure 3d presents the surface PEDOT‐to‐PSS ratio extracted from the deconvolution of the S(2p) spectra (Figure S10), showing an increasing tendency after laser irradiation and a further enhancement following water treatment. This trend indicates an enrichment of PEDOT at the film surface relative to the pristine state. To determine whether this surface enrichment arises from chemical loss or degradation of PSS, bulk‐sensitive UV–vis spectroscopy was performed in the 200–300 nm range (Figure 3e), where absorption features are primarily attributed to PSS [71, 73]. Notably, these spectral features remain essentially unchanged across pristine, L‐, and W‐state samples, in contrast to previous reports involving chemical depletion of PSS [64, 74].

Considering the large difference in probing depth between surface‐sensitive XPS (∼nm) [75] and bulk‐sensitive UV–vis spectroscopy (∼µm) [76], together with the prior removal of excess PEDOT:PSS during the interlocking process, these observations suggest that the increased surface PEDOT‐to‐PSS ratio originates from physical rearrangement rather than chemical modification of the polymer components [77]. In line with this interpretation, laser irradiation is considered to promote a preferential redistribution of PEDOT‐rich domains toward the surface, where photothermal effects are most pronounced, while PSS‐rich regions are relatively displaced toward the film interior. This interpretation is supported by the cross‐sectional phase image of the L‐state shown in Figure S12, consistent with thermally induced structural evolution reported for PEDOT:PSS systems [39, 64, 65]. Upon subsequent water exposure and drying, enhanced chain mobility [13, 78] enables further redistribution of the polymer components along the spatial gradient established by laser irradiation, resulting in an increased surface compositional contrast, as suggested by surface phase images of L–W transition presented in Figure S8.

The role of hydration in enabling reversible state switching was further examined using FT‐IR spectroscopy in attenuated total reflectance (ATR) mode (Figure 3f). Upon laser irradiation, the broad O–H stretching band in the 3200–3640 cm^−^^1^ region [79, 80] is noticeably suppressed in both L‐F and L‐S samples, indicating a reduction in hydrogen‐bonded water within the PEDOT:PSS matrix. In addition, subtle spectral changes in the 1300–1600 cm^−1^ region are consistent with laser‐induced reorganization of the conjugated PEDOT backbone [81] identified by Raman analysis, with slightly more pronounced C–H intensity [82] changes under the slow‐scan condition.

After water exposure and drying, the O–H band largely recovers in the W‐state, with more complete restoration in W‐F compared to W‐S. Importantly, no new IR bands associated with irreversible chemical modification are detected, confirming that the laser‐water process primarily modulates hydration and intermolecular interactions rather than inducing permanent chemical reactions.

**FIGURE 4:**
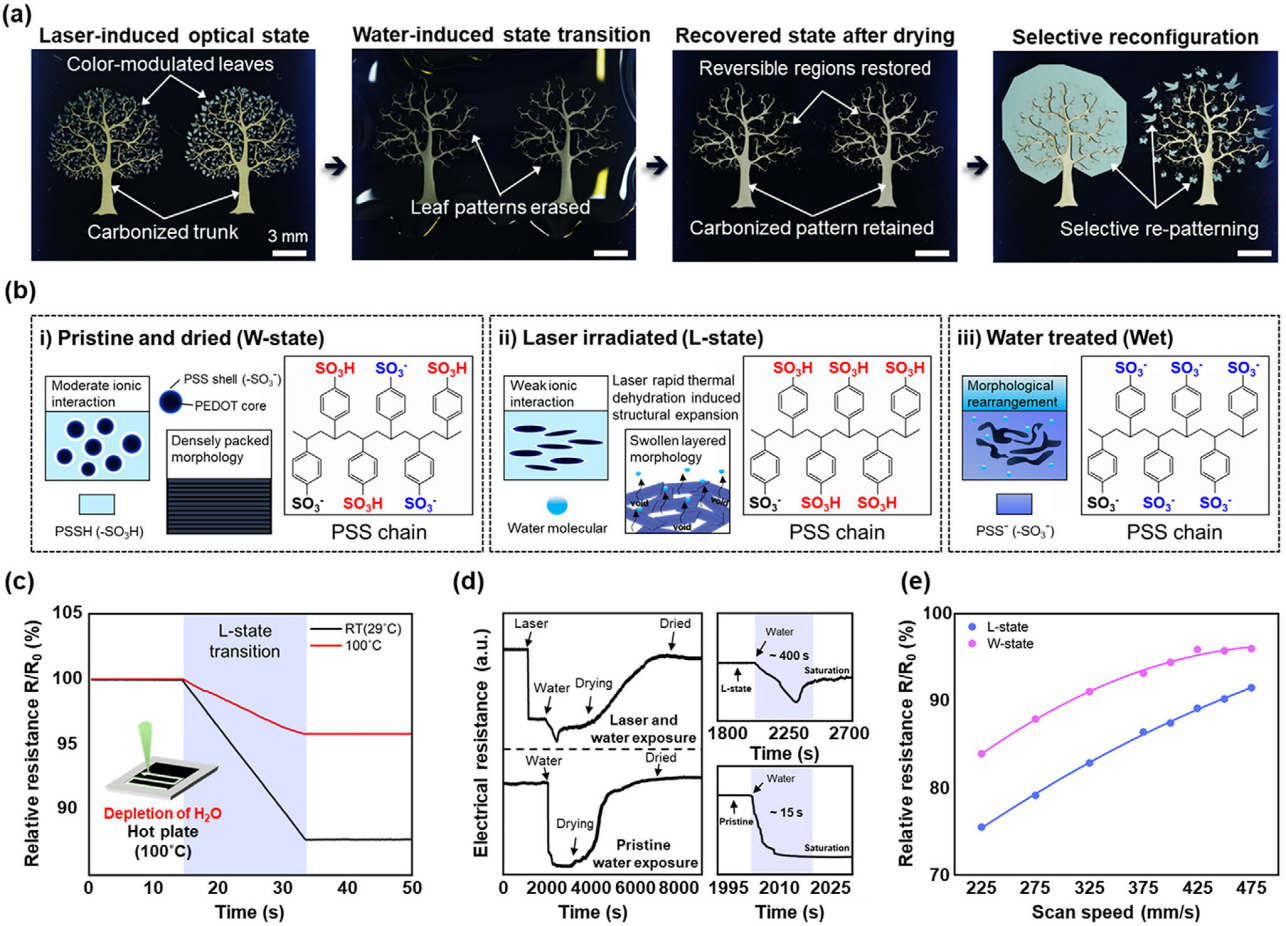
Electrical response during hydration‐driven L–W state switching. (a) Spatially selective optical state switching in a single PEDOT:PSS film, where water exposure restores blue‐transitioned regions while carbonized patterns remain unchanged. (b) Schematic illustration of the proposed state‐switching mechanism, involving reversible hydration of PSS chains adjacent to PEDOT^+^. (c) Real‐time electrical resistance response during laser irradiation under different temperature conditions, highlighting hydration‐dependent electrical state evolution. (d) Time‐dependent electrical resistance changes of pristine and L‐state regions during hydration. (e) Relative electrical resistance of L‐ and W‐states normalized to the pristine state as a function of laser scanning speed (15 mW laser power).

The spatial selectivity and dynamic nature of the laser‐water‐mediated state switching are demonstrated in Figure 4a. By controlling the laser irradiation conditions, blue‐transitioned leaf patterns (15 mW–475 mm s^−1^) and permanently carbonized trunks (15 mW–25 mm s^−1^) can be selectively written within a single PEDOT:PSS film. Upon water exposure, only the non‐carbonized, blue‐transitioned regions are erased, while the carbonized patterns remain intact. Subsequent laser irradiation enables selective re‐patterning of the erased regions, confirming that reversible and irreversible states can be spatially encoded and independently addressed within the same film.

The underlying mechanism of this selective reversibility is illustrated schematically in Figure 4b. The proposed state switching mechanism is governed by the reversible hydrogen‐bond interactions associated with PSS, as described by the following equation [13, 14], excluding the sulfonate groups that serve as counterions for PEDOT^+^ [13].

$$\begin{array}{cc} & \mathrm{Hydration}:\;\;\;\mathrm{PSS}\left(-S{O_{3}}^{-}\right)+H_{3}O^{+}\\ & ↔\mathrm{Dehydration}:\;\mathrm{PSS}\left(-SO_{3}H\right)+H_{2}O \end{array}$$

In the dried pristine state, electrostatic interactions between PEDOT^+^ and deprotonated PSS^−^ stabilize a granular morphology. Laser irradiation locally induces rapid heating [83] and dehydration [32], weakening ionic interactions and promoting morphological rearrangement toward a swollen and interconnected nanofibrillar structure [57], accompanied by partial protonation of PSS, which stabilizes the modified structure. Subsequent water exposure rehydrates the PSS network, restores deprotonation, and increases polymer chain mobility, thereby enabling structural relaxation and recovery of electrostatic interactions toward the W‐state. Notably, the L–W transition also occurs under high‐humidity conditions without direct water immersion, emerging above ∼70% relative humidity (Figure S13), further supporting a hydration‐driven reorganization mechanism consistent with prior reports on PEDOT:PSS [14, 78].

To isolate the role of water in hydration‐mediated state changes, electrical resistance was monitored in real time during laser irradiation under different hydration conditions (Figure 4c), providing a functional readout of the evolving material state. Under ambient conditions (RT, 29°C), laser irradiation leads to a continuous decrease in relative resistance during the L‐state transition window. A similar resistance decrease is also observed when irradiation is performed at 100°C on a hot plate. Such resistance reduction upon laser is consistent with a previous report on thermally processed PEDOT:PSS [64]. However, the magnitude of the resistance change at 100°C is markedly reduced, indicating that diminished hydration under elevated‐temperature conditions suppresses the extent of laser‐induced electrical state development.

The kinetics of electrical state switching during water exposure and subsequent drying are further resolved by real‐time resistance measurements shown in Figure 4d. Upon water exposure, the pristine interlocked PEDOT:PSS film exhibits a rapid resistance decrease with a short saturation time (∼15 s). In contrast, L‐state regions show a slower, non‐monotonic response, with an initial resistance decrease upon water exposure, an intermediate recovery regime, and a long‐time relaxation occurring over several hundred seconds (∼400 s). This distinct difference indicates that, unlike the rapid optical transition (Movie S1), electrical state switching is governed by slower, molecular‐scale reorganization processes.

Figure 4e summarizes the scan‐speed‐dependent electrical resistance of the L‐state regions and the corresponding W‐state regions, presented as relative resistance normalized to the pristine state. In the L‐state, the electrical resistance decreases monotonically with decreasing scan speed, consistent with progressively stronger laser‐induced modification under higher thermal doses. After water treatment, the resistance increases relative to the L‐state, reflecting partial relaxation of the laser‐defined electrical state. Notably, the W‐state resistance remains dependent on the initial laser processing conditions, indicating that hydration does not simply restore the pristine state but instead drives relaxation along pathways determined by the laser‐defined configuration. To clarify this relaxation behavior, repeated laser‐water‐induced state switching over multiple cycles was further investigated, as shown in Figure S14. The observed evolution of both morphology and electrical resistance, before stabilization, suggests that hydration‐driven relaxation proceeds through history‐dependent reorganization within the PEDOT:PSS system. This behavior further supports that the W‐state represents a nontrivial relaxation of the L‐state rather than a simple reversal process. After stabilization, both optical and electrical properties exhibit reversible switching between L‐ and W‐states, consistent with reconfigurable coloration and modulation of ionic interactions between PEDOT and PSS induced by laser‐water stimulation.

## 3 Conclusion

In this work, we investigated laser‐water‐mediated state switching in PEDOT:PSS from the perspective of material state definition and relaxation. Using a mechanically interlocked PEDOT:PSS configuration that maintains film integrity under repeated laser irradiation and hydration, we systematically examined how laser‐defined states evolve upon subsequent water exposure. Continuous‐wave laser irradiation locally defines distinct optical, morphological, and molecular states without inducing irreversible chemical modification, while subsequent hydration drives their relaxation rather than simple erasure. Correlative analyses of optical response, micro‐ and nanoscale morphology, spectroscopic signatures, and electrical behavior reveal that hydration does not necessarily restore the pristine state but instead promotes relaxation pathways determined by the initial laser‐defined configuration. These results demonstrate that laser irradiation creates metastable structural states in PEDOT:PSS, whose relaxation behavior under hydration depends strongly on the prior processing conditions.

Overall, this study establishes that water functions not merely as a reset stimulus but as an active mediator governing the relaxation of laser‐defined structural states in PEDOT:PSS. Because the final relaxed state depends on both the initial laser‐defined configuration and the subsequent hydration conditions, the system exhibits history‐dependent behavior under sequential stimuli. This finding highlights new opportunities for designing conductive polymer systems whose structural and electrical responses encode prior processing or environmental exposure, and for engineering programmable relaxation pathways beyond conventional instantaneous stimulus–response mechanisms.

## 4 Experimental Section

### 4.1 Optical Set‐Up

A 532 nm CW laser (3 W, Arktis Laser, Canada) was used for the state switching process. A half‐wave plate (HWP) and polarizing beam Splitter (PBS) were used to control the laser power. A computer‐controlled galvano‐mirror scanning system (hurrySCAN II 14, Scanlab, Germany) integrated with an F‐theta lens (4401‐547‐000‐26, Excelitas Technologies, USA) was used for laser scanning. A schematic illustration of the optical setup used for laser irradiation and scanning is provided in Figure S15. The laser spot diameter on the sample surface was approximately 10 µm (1/e^2^), as estimated from the optical configuration assuming a Gaussian beam profile.

### 4.2 Materials and Laser Interlocking PEDOT:PSS on PU Substrate

Polyurethane acrylate varnish (SEA‐1, CCTech, South Korea) was spin‐coated on a glass substrate for 15 s at 500 rpm, followed by UV irradiation to induce photocrosslinking from PUA to PU. PEDOT:PSS aqueous solution (Clevios PH‐1000, Heraeus, Germany) was drop‐cast onto PU‐coated glass (0.0625 mL cm^−2^) and dried on a hot plate at 40°C.

For stable laser interlocking, a 532 nm continuous‐wave laser was applied from the backside of the PEDOT:PSS‐coated region at a scan speed of 450 mm s^−1^ with a fixed laser power of 60 mW and a scanning interval of 10 µm.

The thickness of the laser‐interlocked PEDOT:PSS film was approximately 6 µm. Water treatment was performed by immersing the samples in deionized water at room temperature for ∼600 s, followed by drying under ambient conditions. Unless otherwise specified, this water treatment refers to post‐laser hydration applied to laser‐processed samples to induce state relaxation.

### 4.3 Optical, Morphological, Compositional, Chemical, and Electrical Characterization

For morphological, compositional, chemical, and electrical characterization, all samples were laser scanned with a 10‐µm hatch spacing. Optical microscopy imaging was conducted using an optical microscope (BX53M, Olympus, Japan) under ambient light. SEM (VEGA, TESCAN, Czech Republic) and HRKV‐SEM (JSM‐7900F, JEOL, Japan) were used to investigate surface and cross‐sectional microstructure. Surface topography and phase images were obtained using an atomic force microscope (XE‐7, Park Systems Inc., South Korea). A Raman spectrometer (Ramantouch 532 nm, Bruker, USA) was used to analyze the molecular structure. X‐ray diffractometer (X'pert^3^ Powder, Malvern Panalytical, Netherlands) was used to analyze the crystalline structure. X‐ray photoelectron spectroscope (AXIS SUPRA, Kratos Analytical, UK) and UV–vis spectrometer (V‐770, Jasco, Japan) were used to investigate compositional changes during state switching. FT‐IR spectrometer (IS50 ATR, Thermo Fisher Scientific, USA) was used to examine chemical bonding changes. To measure electrical conductivity, a constant voltage of 0.05 V was applied using a source meter (source measure unit 2450, Keithley Instruments, USA). Electrical measurements were performed using a two‐probe configuration with a fixed probe spacing of 20 mm.

## Author Contributions

**Won‐Seok Kim**: investigation, data curation. **Chanwoo Kim**: investigation, formal analysis, data curation, writing – original draft, methodology, visualization. **Songkil Kim**: investigation, validation, methodology. **Habeom Lee**: resources, writing – review and editing, writing – original draft, conceptualization, validation, funding acquisition, supervision. **Sanghun Lee**: investigation, validation. **Soonwook Jung**: investigation, validation.

## Conflicts of Interest

The authors declare no conflicts of interest.

## Supporting information




**Supporting File 1**: smll74614‐sup‐0001‐SuppMat.docx.

**Supporting File 2**: smll74614‐sup‐0002‐MovieS1.mp4.

## Data Availability

The data that support the findings of this study are available from the corresponding author upon reasonable request.
