# Supplementary material for: Laser‐Defined States and Hydration‐Driven Relaxation in PEDOT:PSS
Source: Small. 2026 Jul 28;22(51):e74614. doi: 10.1002/smll.74614 (PMC13569062; doi:10.1002/smll.74614)
Supplement: Supplementary file 1 [file SMLL-22-e74614-s001.docx]

Supporting Information

**Laser-Defined Material States and**

**Hydration-Driven Relaxation in PEDOT:PSS**

*Chanwoo Kim, Soonwook Jung, Won-Seok Kim, Sanghun Lee, Songkil Kim, Habeom Lee*^1^*

^1^ School of Mechanical Engineering, Pusan National University, Busan 46241, Republic of Korea

* Corresponding authors: hblee@pusan.ac.kr (Prof. H. Lee).

**Figure S1.** Digital images of laser patterned tree on PEDOT:PSS showing blue optical transition region and carbonized region which were processed at different specific laser conditions, respectively. Under mechanical peeling test, blue optical transition region showed durability while carbonization region left residue on the scotch tape.

**Figure S2.** Laser backside scanned PEDOT:PSS through a PU substrate. (a) Optical microscopy images showing the interface between PEDOT:PSS and the PU substrate of laser treated region and the corresponding region after water treatment. (b) Cross-sectional SEM images of the interface in laser treated area before and after water treatment. The formation of wavy structures indicates strong interlocking at the interface between PEDOT:PSS and the PU substrate.^[1]^

**Figure S3.** Laser power versus scan speed window representing laser-induced state change. L-state, defined as the color-changed non-carbonized state, does not include the mixed state.

**Figure S4.** Laser-induced structural coloration. (a) Schematic illustration showing the origin of optical diffraction. (b) Digital images revealing scan-angle-dependent, (c) viewing-angle-dependent and (d) hatch-spacing-dependent optical responses of L-state region under ambient white LED light.

**Figure S5.** The partially carbonized state exhibits carbonized features at the center and swollen regions at the rim, arising from Gaussian intensity profile of the laser. This observation indicates that excessive thermal input, beyond the threshold required to induce structural expansion, leads instead to carbonization of the material and contributes to the grayish-blue coloration observed in L-state and the gray coloration observed in W-state. (a) Optical microscopy images, cross-sectional height profiles, and (b) SEM images of the mixed state in L-state and W-state are presented. The carbonized features largely retain their morphology after water treatment, except for isotropic shrinkage associated with the relaxation of the swollen region. This behavior distinguishes the laser-defined blue transition state from irreversible material degradation.

**Figure S6.** SEM images of L-state under 15 mW at different scan speed. (a) undulating surface morphology region. (b) dome-like largely uniform surface morphology region.

**Figure S7.** AFM topography images of W-state under 15 mW at various scan speeds.

**Figure S8.** (a) Optical images showing sequence of L- to W-state transition via capillary driven water absorption, (b) its height profile and (c) AFM phase images suggesting surface redistribution of PEDOT:PSS components. Considering that brighter regions are likely to be richer in PEDOT domains, the observed evolution of phase contrast is consistent with the possibility of depth-scale rearrangement of conductive polymer components during state switching.

**Figure S9.** (a) Schematic illustration of continuous-wave laser induced heat field. (b) AFM phase images of L-state showing symetrically aligned features of PEDOT:PSS domains which follows laser induced thermal energy field.

**Figure S10.** XPS analysis for sulfur(2p) atoms of pristine, L-F, L-S, W-F and W-S. Peaks around 168 and 164 eV were originated from PSS and PEDOT, respectively.^[2]^

**Figure S11.** UV-vis absorbance spectrum from 300 nm to 1100 nm for pristine, L-F, L-S, W-F and W-S. Most of absorbance derived from PEDOT and represent oxidation state of PEDOT. Specifically, the increase at around 900 nm represents polaron doped state of PEDOT after laser irradiation.^[3]^ The near-infrared region is shown for completeness; abrupt intensity variations originate from instrumental saturation and are not considered in the discussion.

**Figure S12.** (a) Experimental setup for cross-sectional AFM imaging of L-state within PEDOT:PSS. (b) Schematic illustration of the AFM investigated region within the cross-section of L-state. (c) Cross-sectional AFM phase image of L-state. Considering that brighter regions are likely to be richer in PEDOT domains, the observed phase contrast suggests the possibility of depth-dependent rearrangement of polymer components.

**Figure S13.** After 30 min exposure to water vapor in a humidity chamber: (a) optical images of the L-state under different humidity conditions and (b) corresponding line-width profiles.

**Figure S14.** Repeated laser-water-induced state switching over 10 cycles under a laser scan condition of 15 mW - 475 mm/s. (a) Optical images of L-state and W-state at 1^st^, 4^th^, 7^th^, 10^th^ cycles, showing the structural expansion behavior in L-state and the structural partial relaxing behavior in W-state during repeated state switching. (b) Changes in line width of L-state during repeated L-state transitions, showing progressively increasing configuration until becoming stabilized. (c) Evolution of the relative resistance in L-state and W-state during repeated state switching. After stabilization, the relative resistance exhibits reversible switching depending on the state of PEDOT:PSS (hatch size: 10 μm). These observations support the history-dependent structural and electrical evolution, and indicate reconfigurable optical and electrical state switching in hydration-sensitive conductive polymers.

**Figure S15.** Schematic diagram of the optical system used in the laser process

**References**

1.	Won, D.; Kim, H.; Kim, J.; Kim, H.; Kim, M. W.; Ahn, J.; Min, K.; Lee, Y.; Hong, S.; Choi, J., *Nature Electronics* **2024,** *7* (6), 475-486. DOI <https://doi.org/10.1038/s41928-024-01161-9>.

2.	Won, D.; Kim, J.; Choi, J.; Kim, H.; Han, S.; Ha, I.; Bang, J.; Kim, K. K.; Lee, Y.; Kim, T.-S., *Science Advances* **2022,** *8* (23), eabo3209. DOI <https://doi.org/10.1126/sciadv.abo3209>.

3.	Wang, X.; Feng, Y.; Sun, K.; Chai, N.; Mai, B.; Li, S.; Chen, X.; Zhao, W.; Zhang, Q., *Energy & Environmental Materials* **2024,** *7* (3), e12650. DOI <https://doi.org/10.1002/eem2.12650>.
